# Inhibition of CYP3A-mediated Midazolam Metabolism by *Kaempferia Parviflora*

**DOI:** 10.14252/foodsafetyfscj.D-21-00013

**Published:** 2022-03-03

**Authors:** Yumika Kashiwabuchi, Yuki Nishimura, Norimitsu Kurata, Mariko Iwase, Yuji Kiuchi, Koji Nobe

**Affiliations:** 1 Department of Pharmacology, Showa University School of Pharmacy, 1-5-8 Hatanodai, Shinagawa-ku, Tokyo 142-8555, Japan; 2 Department of Pharmacology, Showa University School of Medicine, 1-5-8 Hatanodai, Shinagawa-ku, Tokyo 142-8555, Japan; 3 Pharmacological Research Center, Showa University, 1-5-8 Hatanodai, Shinagawa-ku, Tokyo 142-8555, Japan

**Keywords:** 5, 7-dimethoxyflavone, CYP3A, Cytochrome P450, food-drug interactions, *Kaempferia parviflora*, time-dependent inhibition

## Abstract

*Kaempferia parviflora* (KP) extract has recently attracted attention in Japan as a dietary supplement; however, there is little information regarding food-drug interactions (FDIs). The current study was conducted to clarify the FDI of KP extract via inhibition of cytochrome P450 3A (CYP3A), a typical drug-metabolizing enzyme. The inhibitory effects of KP extract and its main ingredients, 5,7-dimethoxyflavone (5,7-DMF) and 3,5,7,3’,4’-pentamethoxyflavone (3,5,7,3’,4’-PMF), on CYP3A-mediated midazolam 1’-hydroxylation (MDZ 1’-OH) activity were investigated in human liver microsomes. In addition, the effect of a single oral treatment with KP extract (135 mg/kg) on oral MDZ (15 mg/kg) metabolism was investigated in rats. Serum MDZ concentration was analyzed and pharmacokinetic parameters were compared with the control group. KP extract competitively inhibited MDZ 1’-OH activity with an inhibition constant value of 78.14 µg/ml, which was lower than the estimated concentration in the small intestine after ingestion. Furthermore, KP extract, 5,7-DMF, and 3,5,7,3’,4’-PMF inhibited the activity in a time-, NADPH-, and concentration-dependent manner. *In vivo* study showed that administration of KP extract to rats 2 h before MDZ significantly increased the area under the serum concentration-time curve and the maximum concentration of MDZ significantly by 2.3- and 1.9- fold, respectively (*p *< 0.05). Conversely, administration of MDZ 18 h after KP extract treatment displayed a weaker effect. These results suggest that KP extract competitively inhibits CYP3A-mediated MDZ metabolism, and that this inhibition may be time-dependent but not irreversible. This work suggests an FDI through CYP3A inhibition by KP extract.

## 1. Introduction

Dietary supplements are widely used to maintain and improve health and beauty. In recent years, self-medication has been promoted in Japan in response to the increase in medical expenses in an aging society with a declining birthrate, and the market for supplements is expected to expand further in the future. Among them, products using herbal extracts are classified as foods in Japan, but some products, such as St. John’s wort, saw palmetto, and echinacea, are used as drugs in European countries^1^^,^^2^^,^^3^^)^. In Japan, approval review regulations for herbal medicinal products were announced in 2007, and red vine leaf extract and chest berry extract have been approved as direct over-the-counter (OTC) medicines. Since these OTC drugs and supplements derived from herbal extracts are easily available at drug stores and online retailers, the number of people using them has been increasing.

However, it is very important to pay attention to drug interactions in order to safely use drugs. In particular, drug interactions mediated by inhibition of the typical drug-metabolizing enzyme, cytochrome P450 3A (CYP3A), is known to be an important factor in the incidence of adverse effects with concomitant drugs. Drug-drug interactions caused by CYP3A inhibition have been reported in many drugs such as simvastatin and nifedipine, and careful attention has been paid to them^4^^,^^5^^)^.

In addition to drug-drug interactions, food-drug interactions (FDIs) have also been reported. As a typical example of such FDIs, grapefruit juice (GFJ) has been shown to inhibit small intestinal CYP3A activity, thereby increasing the blood levels of concomitant drugs metabolized by this enzyme, such as felodipine, triazolam, and simvastatin, and increasing the possibility of adverse effects^6^^,^^7^^,^^8^^)^. Therefore, it is important to pay attention to FDIs to ensure the safe use of these combinations. However, there are limited reports of such FDIs; therefore, in many cases, it is uncertain whether their combined use is safe. In general, dietary supplements tend to be regarded as safe, and physicians and pharmacists rarely pay attention to their combined use with medicines. This can lead to unexpected adverse effects. For these reasons, providing information on FDIs and making it available to physicians, pharmacists, and patients is very important for accurate and safe self- medication.

*Kaempferia parviflora* (KP), also called black ginger or Krachai Dam, belongs to the family Zingiberaceae, and the rhizome has long been used as a folk medicine in Thailand. As a functional ingredient, polymethoxyflavones, including 5,7-dimethoxyflavone (5,7-DMF) and 3,5,7,3’,4’-pentamethoxyflavone (3,5,7,3’,4’-PMF), have been found, and their various pharmacological effects, such as antioxidant, antitumor, and anti-inflammatory activities, have been shown^9^^,^^10^^,^^11^^,^^12^^,^^13^^)^. Moreover, KP extract has recently attracted attention as a dietary supplement in Japan because of its ability to reduce body fat accumulation and improve lipid metabolism and muscle endurance^14^^,^^15^^,^^16^^)^.

Since KP extract may be used by both healthy people and patients taking prescription medications, it is predicted that this supplement will be ingested in combination with drugs. There have been few reports on FDIs through CYP3A inhibition by KP extract. KP extract reportedly inhibited CYP3A activity in an *in vitro* experiment using mouse liver microsomes^17^^)^. In addition, Ochiai et al reported that administration of 5,7-DMF to mice for ten days increased the area under the serum concentration-time curve (AUC) and elimination half-life (t_1/2_) of midazolam (MDZ), which is a probe drug of CYP3A, and decreased hepatic CYP3A expression^18^^)^. However, both reports used mice and whether the same effects will also be observed in humans is unclear. In addition, it is important to investigate the effect of KP extract treatment, which is a mixture of several polymethoxyflavones other than 5,7-DMF, *in vivo*.

Mechanism-based inhibition (MBI) is an important mechanism in drug interactions. In this case, the reactive metabolic intermediate produced by CYP irreversibly inactivates this enzyme, which can lead to long-lasting severe adverse effects. It should be noted that GFJ is a typical example that causes MBI, and thus even foods can cause this severe inhibition^19^^)^. Therefore, it is important to investigate whether KP extract causes MBI of CYP3A, since this enzyme is involved in the metabolism of many clinically important drugs.

Therefore, the current study was conducted to clarify the inhibitory effects of KP extract and its main ingredients, 5,7-DMF and 3,5,7,3’,4’-PMF, on CYP3A-mediated MDZ 1’-hydroxylation in human liver microsomes (HLM). In addition, we examined the effect of KP extract administration on MDZ metabolism in rats *in vivo*. The aim of this study was to investigate FDI through the inhibition of CYP3A by KP extract and to provide information on the safety of KP extract during medication. This information is very important for promoting the proper use of dietary supplements.

## 2. Materials and Methods

### 2.1 Chemicals

MDZ maleate was kindly donated by Nippon Roche Co., Ltd. (Tokyo, Japan). MDZ injection (Dormicum^®^ injection, 10 mg/2 ml) was purchased from Astelas Pharma Inc. (Tokyo, Japan). 1’-Hydroxymidazolam was purchased from Daiichi Pure Chemicals Co., Ltd. (Tokyo, Japan). 5,7-DMF, nitrazepam (NZP), and diazepam were purchased from Wako Pure Chemical Industries, Ltd. (Osaka, Japan). 3,5,7,3’,4’-PMF was purchased from Funakoshi Co., Ltd. (Tokyo, Japan).

A pooled sample of HLM (pooled from 10 donors) was purchased from the Non-Profit Organization Human and Animal Bridging Research Organization (Chiba, Japan). All other chemicals used were of analytical grade.

### 2.2 Preparation of *Kaempferia Parviflora* extract and Analysis of Polymethoxyflavones

The dried rhizomes of KP (Maechu Co., Ltd., Nara, Japan) were extracted twice with 80% ethanol. After filtration, the solution was evaporated under vacuum to yield dry powder.

**Fig. 1.**
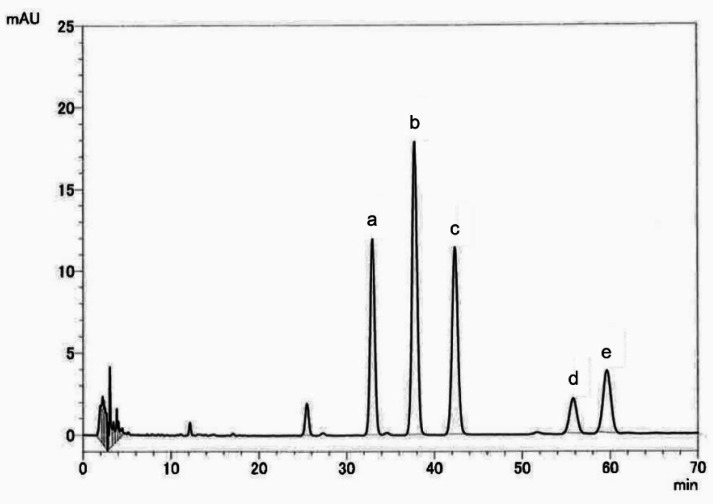
HPLC chromatogram of KP extract. KP extract (5 µg) was injected onto HPLC. a, 3,5,7,3’,4’-PMF, b, 5,7-DMF, c, 5,7,4’-trimethoxyflavone, d, 3,5,7-trimethoxyflavone and e, 3,5,7,4’-tetramethoxyflavone.

The contents of 5,7-DMF and 3,5,7,3’,4’-PMF in the extract were analyzed by high-performance liquid chromatography (HPLC) equipped with a CAPCELL PAK C18 UG120 column (4.6 mm × 250 mm, Shiseido, Tokyo, Japan) with a UV detector at 260 nm. The mobile phase was 1% formic acid: acetonitrile (70:30), and the flow rate was 1.0 ml/min. The HPLC chromatogram of the KP extract is shown in Fig. 1. The total amount of polymethoxyflavones (3,5,7-trimethoxyflavone, 3,5,7,4’-tetramethoxyflavone, 5,7,4’-trimethoxyflavone, 5,7-DMF, and 3,5,7,3’,4’-PMF) in the extracted dry powder was 27.5%, in which 5,7-DMF and 3,5,7,3’,4’-PMF were present at 7.45% and 7.2%, respectively.

### 2.3 Direct Inhibition Study

An *in vitro* study was carried out to investigate the inhibitory effects of KP extract, 5,7-DMF, and 3,5,7,3’,4’-PMF on CYP3A-mediated MDZ 1’-hydroxylation (MDZ 1’-OH) activity.

The reaction mixtures, each with a total volume of 200 µL, consisting of 100 µg pooled HLM, 10 µM MDZ, and a nicotinamide adenine dinucleotide phosphate (NADPH)-generating system (1 mM NADPH, 8 mM glucose 6-phosphate, and 0.5U glucose 6-phosphate dehydrogenase) in 80 mM phosphate buffer (pH 7.4), were incubated at 37°C for 5 min in the presence of KP extract (40-80 µg/ml), 5,7-DMF (0.03-30 µM), or 3,5,7,3’,4’-PMF (0.02-20 µM). The reactions were quenched by the addition of cold methanol: acetonitrile (35:21), followed by the addition of NZP (200 ng) as an internal standard. After centrifugation at 20,800 × *g* for 10 min at 4°C, the supernatant was injected into an HPLC apparatus equipped with a CAPCELLPAK C18 SG120 column (4.6 mm × 250 mm, Shiseido) and 1’-hydroxymidazolam was analyzed according to a previous report^20^^,^^21^^)^. Control activity was determined under the same conditions with a reaction mixture containing water, 1% methanol, or 1% acetonitrile instead of KP extract, 5,7-DMF, or 3,5,7,3’,4’-PMF. Results were expressed as a percentage of each control activity. The inhibition mode and inhibition constant (K_i_) values of KP extract on MDZ 1’-OH activity were calculated using nonlinear regression analysis using GraphPad Prism 7.03 (GraphPad Software Inc., La Jolla, CA, USA).

### 2.4 Pre-incubation Study

Pre-incubation studies were performed to determine whether the metabolic intermediates produced by KP ingredients cause an MBI that inactivates CYP3A. KP extract (120 µg/ml), 5,7-DMF (30 µM), or 3,5,7,3’,4’-PMF (20 µM) was pre-incubated with HLM at 37°C for 10, 20, or 30 min, in the presence or absence of a NADPH-generating system. After the pre-incubation step, MDZ was added, and MDZ 1’-OH activity was assayed as described above.

In addition, another assay was designed to minimize the direct inhibition of CYP3A by KP ingredients by diluting the contents of the inactivation mixture 10-fold. As a primary incubation mixture, KP extract (30-120 µg/ml), 5,7-DMF (6-30 µM), or 3,5,7,3’,4’-PMF (5-20 µM) were pre-incubated in the presence of HLM and a NADPH-generating system at 37°C. Pre-incubation times were 5, 10, 15, or 20 min for KP extract, and 5, 10, 20, or 30 min for 5,7-DMF and 3,5,7,3’,4’-PMF. At determined time points, an aliquot of 50 µL of the primary reaction mixture was moved to 450 µL of the secondary incubation mixture containing 10 µM MDZ, and further incubated for 5 min. The oxidation of MDZ was analyzed as described above.

The decrease in the natural logarithm of the % residual MDZ 1’-OH activity against pre-incubation time was plotted for each inhibitor concentration, and the apparent inactivation rate constant (K_obs_) values were described as the negative slopes of the lines. The maximal inactivation rate constant (k_inact_) and the inhibitor concentration causing half-maximal inactivation (K_I_) were estimated by double-reciprocal plot of the K_obs_ against the inhibitor concentration. The efficiency of inactivation was calculated as the ratio of k_inact_ to K_I_.

### 2.5 Animal Treatment and Sampling

Male Sprague-Dawley rats (Japan Laboratory Animals, Inc., Tokyo, Japan), weighing 200-230 g, were used in this study. The rats were housed in a temperature-controlled room (23°C) with a 12 h light (6:00 AM-6:00 PM) and dark (6:00 PM-6:00 AM) cycle and fed standard laboratory chow ad libitum. The rats were fasted overnight (after 6:00 PM) before sampling. All procedures were reviewed and approved by the Institutional Animal Care and Use Committee of Showa University (Permit Number: 07002, approval date: April 1, 2017) and complied with the guidelines established by the National Institute of Health (NIH) in the Guide for the Care and Use of Laboratory Animals (8th edition, ILAR-NRC).

The rats were divided into three groups. A single KP extract (135 mg/kg) was orally administered to rats (n = 3-5) 2 (9:00 AM) or 18 h (5:00 PM) before oral administration of MDZ (15 mg/kg) (11:00 AM). The equivalent doses of 5,7-DMF and 3,5,7,3’,4’-PMF, calculated from the percentages of these constituents in KP extract, were 19 and 18 mg/kg, respectively. Control rats were administered water 2 h before MDZ administration. Blood samples (200 µL) were collected from the jugular vein before and 15, 30, 45, 60, 90, 120, 180, and 240 min after MDZ administration. The obtained blood samples were centrifuged at 1,600 × *g* for 10 min at 4°C, and the resulting serum samples were stored at -80°C until analysis.

### 2.6 Determination of Serum MDZ Concentration and Pharmacokinetic Analysis

The serum concentrations of MDZ were determined using previously described HPLC methods with slight modifications^22^^,^^23^^,^^24^^)^. Briefly, 100 µL of each serum sample was diluted with 500 µL of 0.1 N NaOH, and 5 ng of diazepam was added as an internal standard. The mixture was extracted with 8 ml of dichloromethane-pentane (1:1) for 1 min and centrifuged at 1,600 × *g* for 10 min. The upper organic solvent phase was transferred to a clean glass tube and evaporated to dryness under a nitrogen stream. The dried residue was dissolved in mobile phase 10 mM acetate buffer (pH 3.8):CH_3_CN:MeOH (50:38:12) at 30°C and injected into HPLC equipment with a CAPCELLPAK C18 MG II column (2.0 × 250 mm, Shiseido). As the mobile phase 50:50 mixture (v/v) of 10 mM phosphate buffer (pH 5.0) and CH_3_CN was used at a flow rate of 0.2 ml/min. The UV detector was set at 220 nm.

The pharmacokinetic parameters of MDZ were assessed by non-compartmental analysis using MOMENT (Excel^®^) based on the moment analytic method^25^^,^^26^^)^. The area under the serum concentration-time curve from time 0 to infinity (AUC_0-∞_) was calculated according to the trapezoidal rule. The maximum serum concentration (C_max_) was obtained from the actual data. The t_1/2_ was calculated by dividing ln 2 by λ, which is the terminal elimination rate constant calculated using a linear regression analysis of at least four points from the terminal portion.

### 2.7 Statistical Analysis

*In vitro* data are expressed as the means of duplicate examinations. *In vivo* data are presented as mean ± standard error (SE). Differences between the groups were analyzed using Dunnett’s test using JMP Pro 15 (SAS Institute Inc., Cary, NC, USA). Differences were considered statistically significant at *p* < 0.05.

## 3. RESULTS

### 3.1 Direct Inhibition Study

**Fig. 2.**
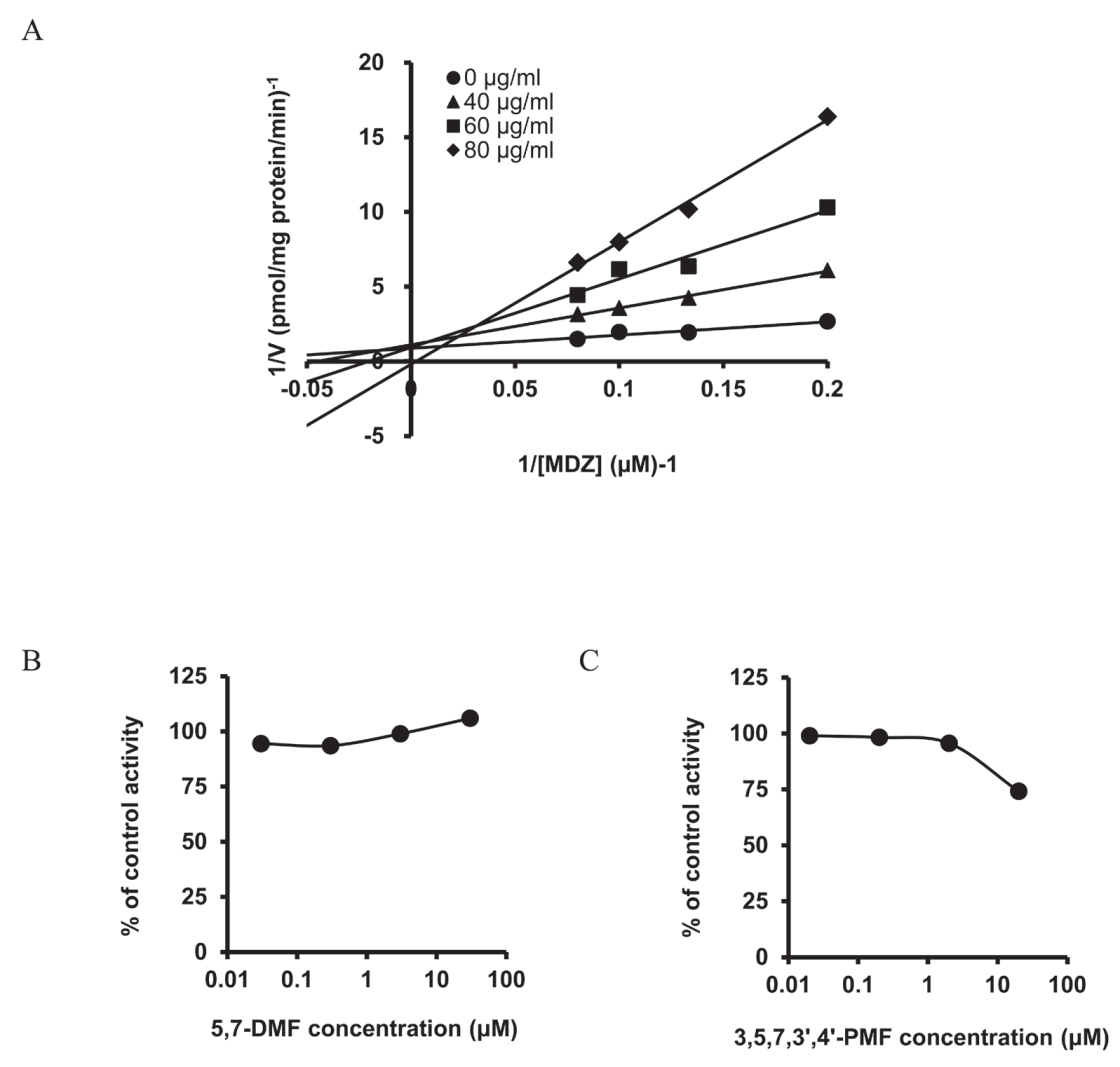
Inhibitory effect of KP extract, 5,7-DMF, and 3,5,7,3’,4’-PMF on MDZ 1’-OH activity in human liver microsomes. KP extract (40-80 µg/ml) (A), 5,7-DMF (0.03-30 µM) (B), or 3,5,7,3’,4’-PMF (0.02-20 µM) (C) were incubated in the incubation mixture containing human liver microsomes (HLM), MDZ (10 µM), and NADPH. Data are shown as the mean of duplicate determinations.

KP extract inhibited CYP3A-mediated MDZ 1’-OH activity competitively with a K_i_ value of 78.14 µg/ml in HLM (Fig. 2A).

In contrast, 5,7-DMF (0.03-30 µM) and 3,5,7,3’,4’-PMF (0.02-20 µM), which are the main polymethoxyflavones in KP extract, did not inhibit MDZ 1’-OH activity even at the high concentrations used in this experiment (Fig. 2B, 2C).

### 3.2 Pre-incubation Study

**Fig. 3.**
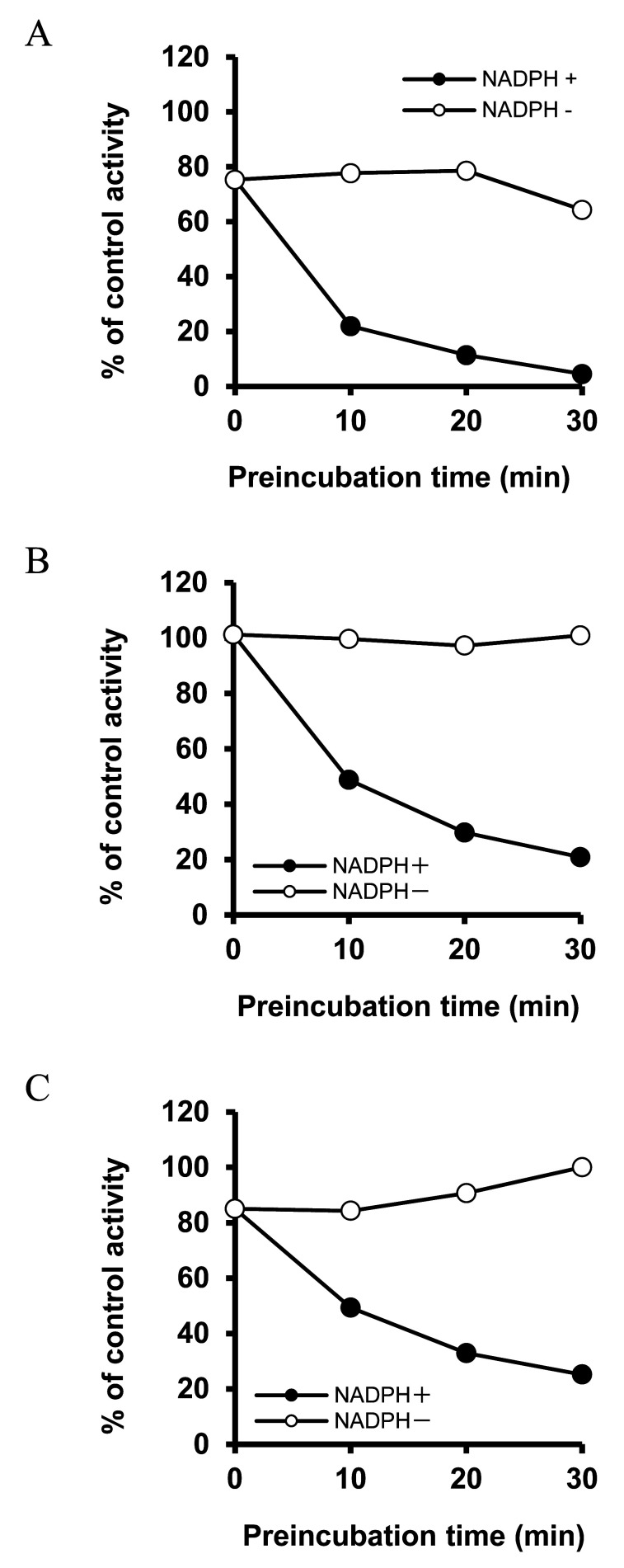
Time- and NADPH-dependent inhibition of MDZ 1’-OH activity induced by KP extract, 5,7-DMF, and 3,5,7,3’,4’-PMF. KP extract (120 µg/ml) (A), 5,7-DMF (30 µM) (B), or 3,5,7,3’,4’-PMF (20 µM) (C) were preincubated with HLM in the presence or absence of NADPH at 37°C for 0-30 min. Following the pre-incubation step, MDZ (10 µM) was added, and the activity was assayed. Data are shown as the mean of duplicate determinations.

**Fig. 4.**
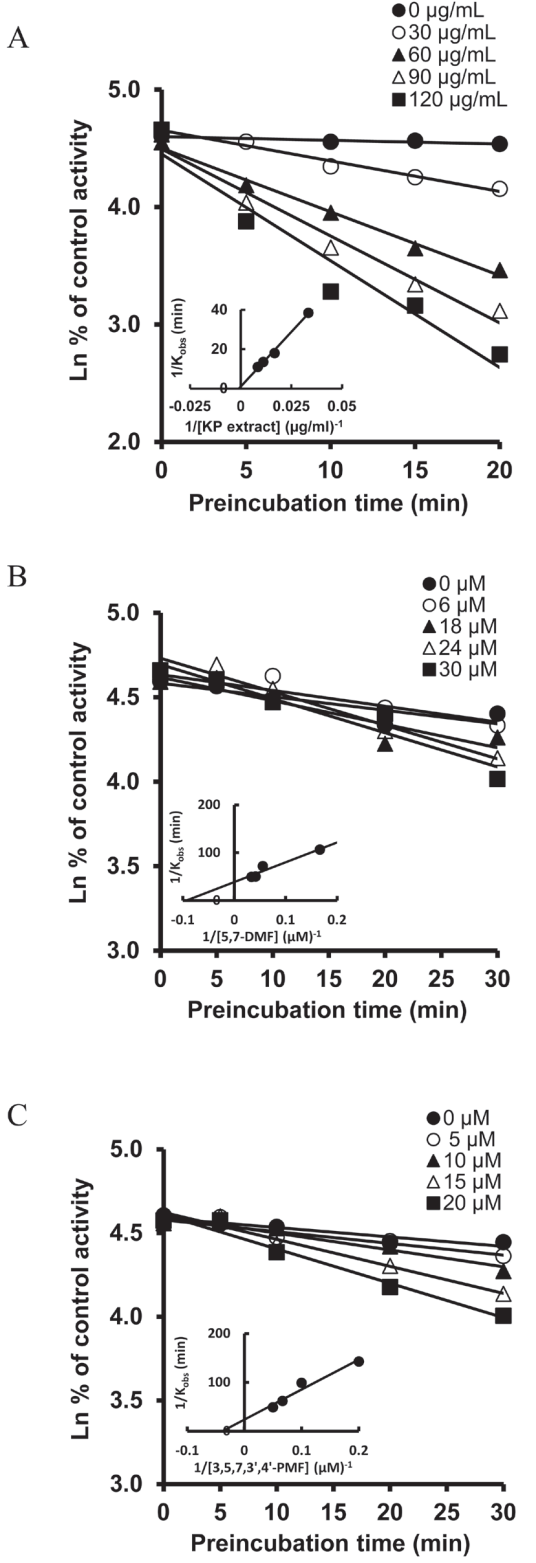
Time- and concentration-dependent inhibition of MDZ 1’-OH activity by KP extract, 5,7-DMF, and 3,5,7,3’,4’-PMF. KP extract (30-120 µg/ml) (A), 5,7-DMF (6-30 µM) (B), and 3,5,7,3’,4’-PMF (5-20 µM) (C) were preincubated with HLM and NADPH at 37°C for 0-30 min. At determined time points, the incubation mixture was diluted 10-fold, MDZ (10 µM) was added, and the activity was assayed. Data are shown as the mean of duplicate determinations.

To determine whether KP extract, 5,7-DMF, and 3,5,7,3’,4’-PMF cause MBI, experiments that included a pre-incubation step were conducted. Pre-incubation with KP extract, 5,7-DMF, and 3,5,7,3’,4’-PMF resulted in a time- and NADPH-dependent decrease in MDZ 1’-OH activity (Fig. 3A, 3B, and 3C). Thereafter, another experiment was performed to analyze the enzyme kinetics for MBI under conditions that minimized the direct inhibition factor by diluting the reaction mixture containing 10 times higher concentrations of KP extract, 5,7-DMF, or 3,5,7,3’,4’-PMF. The results showed that KP extract, 5,7-DMF, and 3,5,7,3’,4’-PMF inhibited MDZ 1’-OH activity in a concentration- and time-dependent manner, and their respective K_I_ values and k_inact_ were as follows: KP extract, K_I_ = 1164 µg/ml and k_inact_ = 1.047 min^-1^; 5,7-DMF, K_I_ = 10.66 µM and k_inact_ = 0.026 min^-1^; 3,5,7,3’,4’-PMF, K_I_ = 25.51 µM and k_inact_ = 0.0016 min^-1^ (Fig. 4A, 4B, and 4C). The k_inact_/K_I_ value of KP extract was 0.00090 ml/min∙µg, and the values of 5,7-DMF, and 3,5,7,3’,4’-PMF were 0.0024 and 0.000063 ml/min∙µmol, respectively.

### 3.3 *In Vivo* study

**Fig. 5.**
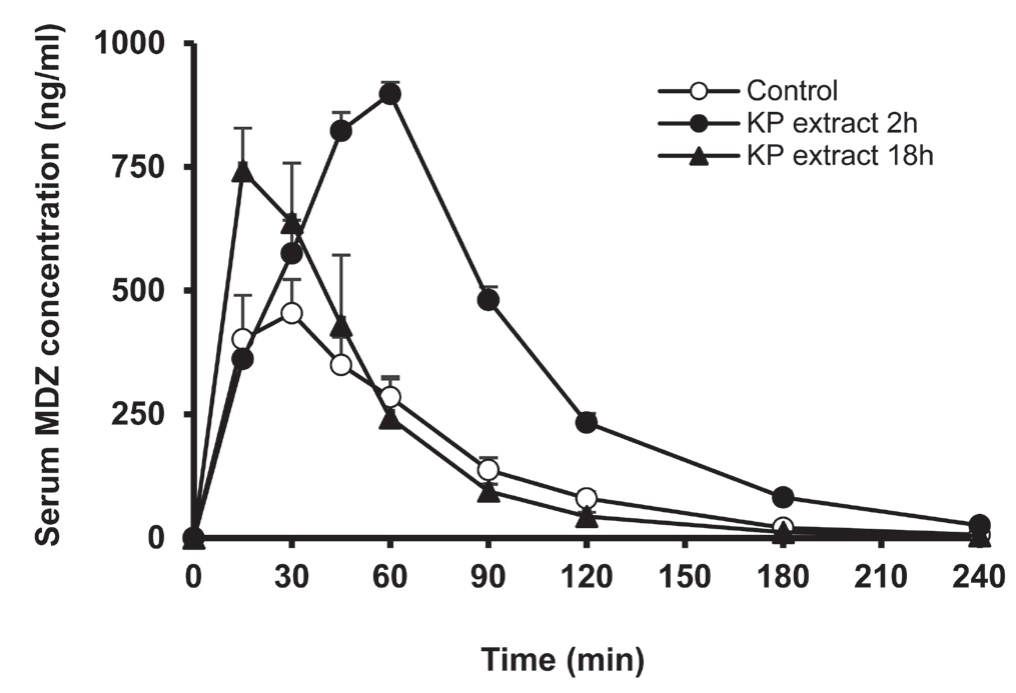
Serum concentration–time profiles of MDZ after a single treatment of KP extract in rats. KP extract (135 mg/kg) or water (control) was orally administered to rats, followed by 2 or 18 h oral administration of MDZ (20 mg/kg). Each point represents the mean ± S.E. of 3-5 rats.

The serum concentration-time profiles and the pharmacokinetic parameters of MDZ after 2 or 18 h of single treatment with KP extract are shown in Fig. 5 and Table 1. In the KP extract treated 2 h before MDZ administration, the mean AUC_0-∞_ and C_max_ of MDZ significantly increased from 564 ± 166 to 1312 ± 55 ng/ml∙h, and from 473 ± 161 to 902 ± 39 ng/ml, respectively (*p *< 0.05), without a change in t_1/2_. KP extract treatment 18 h before MDZ administration showed a significant increase in the C_max_ from 473 ± 161 to 763 ± 148 ng/ml (*p *< 0.05). However, marginal changes were observed in the AUC_0-∞_ and t_1/2_ between the control and treatment groups in this study.

**Table 1.** Pharmacokinetic parameters of MDZ after a single treatment of KP extract in rats

	AUC_0-∞_ (ng/ml∙h)	C_max_ (ng/ml)	t_1/2_ (h)
Control	564 ± 166	473 ± 161	33 ± 3
KP extract 2 h	1312 ± 55*	902 ± 39*	38 ± 10
KP extract 18 h	636 ± 213	763 ± 148*	36 ± 5

Data are shown as the mean ± S.E. for 3-5 rats. * *p* < 0.05, versus control.

## 4. Discussion

In this study, we investigated whether KP extract and its main ingredients, 5,7-DMF and 3,5,7,3’,4’-PMF, cause FDIs mediated by CYP3A inhibition. As a result of the *in vitro* study, KP extract showed competitive inhibition of CYP3A-mediated MDZ 1’-OH activity (Fig. 2A). In addition to the inhibitory effect of KP extract on CYP3A-mediated erythromycin N-demethylation activity reported in mouse liver microsomes^17^^)^, the current study revealed that this inhibition also occurs in HLM.

It is important to predict whether this inhibition can occur *in vivo*. It is unclear how much of the ingredients in the extract that inhibit CYP3A activity are transferred into the blood and liver after oral administration of KP extract to humans. Thus, it is difficult to predict FDI in the liver based on *in vitro* results. CYP3A4 is also highly expressed in the small intestine; therefore, it is important to consider drug interactions in the small intestine^27^^)^. Dietary supplements containing KP extract sold in Japan are labeled with a recommended daily intake of polymethoxyflavones, including 5,7-DMF and 3,5,7,3’,4’-PMF, at 12 mg. Since the KP extract used in this study contained 27.5% polymethoxyflavones, the recommended daily intake was calculated to be 44 mg. The FDA recommends using a volume of 250 mL to calculate the theoretical small intestinal drug concentration after oral administration. According to this, the estimated concentration of KP extract in the small intestine was calculated to be 176 µg/ml by dividing 44 mg by 250 ml. This value is higher than 78.14 µg/ml, which corresponds to the K_i_ value of CYP3A activity obtained in this study, suggesting that KP extract may cause an FDI mediated by CYP3A inhibition, at least in the small intestine.

In contrast, 5,7-DMF and 3,5,7,3’,4’-PMF, the primary polymethoxyflavones of KP extract, did not inhibit MDZ 1’-OH activity (Fig. 2B, 2C). The KP extract used in this study contained 7.45% of 5,7-DMF and 7.2% of 3,5,7,3’,4’-PMF, thus their concentrations in 78.14 µg/ml of KP extract were calculated to be 20.59 µM and 15.08 µM, respectively. None of the compounds inhibited CYP3A activity at this concentration, suggesting that these polymethoxyflavones are not involved in the inhibition of CYP3A activity by KP extract. Therefore, other constituents may be involved in the competitive inhibition of CYP3A activity by KP extract.

Among the mechanisms of CYP inhibition, MBI requires particular clinical attention because the inhibitor irreversibly binds and inactivates CYP, resulting in long-lasting severe inhibition. Therefore, we investigated whether such MBI occurs with KP extract and its main ingredients. As a result of pre-incubation of the reaction mixture containing KP extract, 5,7-DMF, or 3,5,7,3’,4’-PMF, CYP3A4-mediated MDZ 1’-OH activity was inhibited in a time-dependent manner (Fig. 3A, 3B, and 3C). Furthermore, this inhibition was NADPH-dependent, suggesting MBI, in which metabolic intermediates are produced by CYP.

As described above, although 5,7-DMF and 3,5,7,3’,4’-PMF did not inhibit CYP3A activity when added to the reaction mixture at the same time as MDZ, these compounds showed inhibitory effects in this experiment, including the pre-incubation step. From this result, even though the affinity of these compounds for CYP3A is weaker than that of MDZ, the metabolism of co-administered drugs may be inhibited when they are ingested before the administration of drugs that are metabolized by CYP3A, or when they are used in combination with drugs with a lower affinity for CYP3A. 5,7-DMF is reportedly not metabolized by CYP3A4, but by CYP1A1 and CYP1A2, in HLM and recombinant microsomes^28^^)^. Therefore, the metabolic intermediates produced by these CYP enzymes may inactivate CYP3A. To evaluate the risk of time-dependent inhibition, the inactivation efficiency (k_inact_/K_I_) was used^29^^)^. The values of 5,7-DMF and 3,5,7,3’,4’-PMF, obtained from the plots shown in Fig. 4B and 4C, were calculated to be 0.0024 and 0.000063 ml/min∙µmol, respectively. The derived k_inact_/K_I_ of 5,7-DMF for CYP3A was comparable to that of ingredients such as bergamottin and 6,7-dihydroxybergamottin in grapefruit juice, which is known to be a representative inactivator of CYP3A^30^^)^. The above results suggest that KP extract causes MBI of CYP3A, and 5,7-DMF and 3,5,7,3’,4’-PMF are involved in this inactivation.

To clarify whether the inhibition of CYP3A-mediated drug metabolism by the KP extract in *in vitro* experiments could occur *in vivo*, the experiments were performed in rats. To evaluate the direct inhibitory effect of KP extract, a single dose of KP extract was administered 2 h before MDZ administration. The AUC_0-∞_ and C_max_ of MDZ increased 2.3 and 1.9 times, respectively, after treatment with KP extract (Fig. 5, Table. 1). This suggests that KP extract induced drug interactions mediated by CYP3A inhibition in rats *in vivo*. Ochiai et al reported that administration of 5,7-DMF to mice for 10 days resulted in decreased expression of hepatic CYP3A, increased AUC_0-∞_ and C_max_ of MDZ, and prolonged half-life^18^^)^. In contrast, in our experiments, the effect of a single KP extract treatment was examined as a condition that does not affect expression of CYP3A, and the results obtained are considered to be mediated by CYP3A inhibition. From the results of the direct inhibition study *in vitro*, the low contribution of 5,7-DMF and 3,5,7,3’,4’-PMF in CYP3A inhibition was considered. In addition to these polymethoxyflavones, KP extract contains many other ingredients. It is difficult to investigate the inhibitory effect of all these ingredients on CYP3A, but even if the inhibitory ability of each ingredient on CYP3A is weak, it is possible they exhibit inhibitory effects when combined.

Administration of MDZ 18 h after KP extract treatment considering MBI showed that the increase in AUC_0-∞_ and C_max_ of MDZ was less than that after 2 h of MDZ administration. The results suggestive of MBI shown in *in vitro* experiments using HLM and that of this *in vivo* experiment are inconsistent. It has been shown that polymethoxyflavones, such as 5,7-DMF and 3,5,7,3’,4’-PMF contained in KP extract, are distributed mainly in the organs at much higher levels than in blood after oral administration^31^^)^. Therefore, these polymethoxyflavones reached the liver and small intestine and may be involved in the inactivation of CYP3A. Nevertheless, weak inhibition of MDZ metabolism was observed after 18 h. One possible reason for this is that the inhibition mechanisms are time-dependent, but not irreversible. In addition, the species differences in CYP3A need to be considered. Regarding CYP3A isoforms, CYP3A4 is expressed in the human liver and small intestine, whereas in rats, CYP3A2 is expressed in the liver, and other isoforms such as CYP3A9, CYP3A18 and CYP3A62 in the small intestine^32^^)^. These isoforms participate in the metabolism of MDZ, and MDZ is used as an indicator of CYP3A-mediated drug metabolism^33^^,^^34^^)^. However, because of species differences, the degree and the mechanism of CYP3A inhibition by KP extract, 5,7-DMF, and PMF might vary. From the results of *in vivo* experiments examined under the above two conditions, it was considered that in rats *in vivo*, the inhibition of CYP3A by KP extract is caused by a reversible competitive mechanism, and the constituents in KP extract may decrease to less than the concentration that inhibits CYP3A at 18 h after administration.

It is unclear whether KP extract inhibits CYP3A in humans through a competitive or time-dependent mechanism. KP extract is used as a dietary supplement in Japan and may be used in combination with statins when ingested by patients with dyslipidemia to reduce abdominal fat and prevent obesity. Among the statins, simvastatin and atorvastatin are known to be metabolized by CYP3A; therefore, inhibition of this enzyme may increase their levels in the blood, resulting in an increased risk of rhabdomyolysis. KP extract is also used by the elderly population to support muscle endurance. In such cases, it may be used in combination with various drugs, and careful caution is required because CYP3A is involved in the metabolism of many drugs. It is necessary to conduct an optimal study to clarify whether KP extract causes FDIs mediated by CYP3A inhibition in humans.

In conclusion, KP extract showed competitive inhibition of CYP3A-mediated MDZ metabolism in HLM and in rats *in vivo*. In addition, the time-dependent inhibition of MDZ 1’-OH activity was demonstrated by KP extract, 5,7-DMF, and 3,5,7,3’,4’-PMF. These results suggest that KP extract may cause FDIs through CYP3A inhibition. Therefore, this study shows that it is better for patients taking drugs metabolized by CYP3A to avoid using supplements containing KP extract to prevent possible adverse events caused by FDIs.

## References

[1] Zhou S, Chan E, Pan SQ, Huang M, Lee EJD. Pharmacokinetic interactions of drugs with St John’s wort. J Psychopharmacol. 2004; 18(2): 262–276. 10.1177/0269881104042632 15260917

[2] Barry MJ, Meleth S, Lee JY, et al. Effect of increasing doses of saw palmetto extract on lower urinary tract symptoms: a randomized trial. JAMA. 2011; 306(12): 1344–1351. 10.1001/jama.2011.1364 21954478 PMC3326341

[3] Capek P, Šutovská M, Kocmálová M, Fraňová S, Pawlaczyk I, Gancarz R. Chemical and pharmacological profiles of Echinacea complex. Int J Biol Macromol. 2015; 79: 388–391. 10.1016/j.ijbiomac.2015.05.010 25999016

[4] Chauvin B, Drouot S, Barrail-Tran A, Taburet AM. Drug-drug interactions between HMG-CoA reductase inhibitors (statins) and antiviral protease inhibitors. Clin Pharmacokinet. 2013; 52(10): 815–831. 10.1007/s40262-013-0075-4 23703578

[5] Gandhi S, Fleet JL, Bailey DG, et al. Calcium-channel blocker-clarithromycin drug interactions and acute kidney injury. JAMA. 2013; 310(23): 2544–2553. 10.1001/jama.2013.282426 24346990

[6] Lundahl J, Regårdh CG, Edgar B, Johnsson G. Effects of grapefruit juice ingestion - pharmacokinetics and haemodynamics of intravenously and orally administered felodipine in healthy men. Eur J Clin Pharmacol. 1997; 52(2): 139–145. 10.1007/s002280050263 9174684

[7] Lilja JJ, Kivistö KT, Backman JT, Neuvonen PJ. Effect of grapefruit juice dose on grapefruit juice-triazolam interaction: repeated consumption prolongs triazolam half-life. Eur J Clin Pharmacol. 2000; 56(5): 411–415. 10.1007/s002280000156 11009051

[8] Lilja JJ, Kivistö KT, Neuvonen PJ. Grapefruit juice-simvastatin interaction: Effect on serum concentrations of simvastatin, simvastatin acid, and HMG-CoA reductase inhibitors. Clin Pharmacol Ther. 1998; 64(5): 477–483. 10.1016/S0009-9236(98)90130-8 9834039

[9] Yoshino S, Awa R, Miyake Y, et al. Daily intake of *Kaempferia parviflora* extract decreases abdominal fat in overweight and preobese subjects: a randomized, double-blind, placebo-controlled clinical study. Diabetes Metab Syndr Obes. 2018; 11: 447–458. 10.2147/DMSO.S169925 30214264 PMC6120512

[10] Banjerdpongchai R, Suwannachot K, Rattanapanone V, Sripanidkulchai B. Ethanolic rhizome extract from *Kaempferia parviflora* Wall. ex. Baker induces apoptosis in HL-60 cells. Asian Pac J Cancer Prev. 2008; 9(4): 595–600. 19256745

[11] Banjerdpongchai R, Chanwikruy Y, Rattanapanone V, Sripanidkulchai B. Induction of apoptosis in the human Leukemic U937 cell line by *Kaempferia parviflora* Wall.ex.Baker extract and effects of paclitaxel and camptothecin. Asian Pac J Cancer Prev. 2009; 10(6): 1137–1140. 20192599

[12] Tuchinda P, Reutrakul V, Claeson P, et al. Anti-inflammatory cyclohexenyl chalcone derivatives in *Boesenbergia pandurata*. Phytochemistry. 2002; 59(2): 169–173. 10.1016/S0031-9422(01)00451-4 11809452

[13] Sae-wong C, Tansakul P, Tewtrakul S. Anti-inflammatory mechanism of *Kaempferia parviflora* in murine macrophage cells (RAW 264.7) and in experimental animals. J Ethnopharmacol. 2009; 124(3): 576–580. 10.1016/j.jep.2009.04.059 19439175

[14] Lee S, Kim C, Kwon D, Kim MB, Hwang JK. Standardized *Kaempferia parviflora* Wall. ex Baker (Zingiberaceae) extract inhibits fat accumulation and muscle atrophy in *ob/ob* mice. Evid Based Complement Alternat Med. 2018; 1–11. 10.1155/2018/8161042 29997677 PMC5994587

[15] Shimada T, Horikawa T, Ikeya Y, et al. Preventive effect of *Kaempferia parviflora* ethyl acetate extract and its major components polymethoxyflavonoid on metabolic diseases. Fitoterapia. 2011; 82(8): 1272–1278. 10.1016/j.fitote.2011.08.018 21907268

[16] Toda K, Hitoe S, Takeda S, Shimoda H. Black ginger extract increases physical fitness performance and muscular endurance by improving inflammation and energy metabolism. Heliyon. 2016; 2(5): e00115. 10.1016/j.heliyon.2016.e00115 27441286 PMC4946221

[17] Mekjaruskul C, Jay M, Sripanidkulchai B. Modulatory effects of *Kaempferia parviflora* extract on mouse hepatic cytochrome P450 enzymes. J Ethnopharmacol. 2012; 141(3): 831–839. 10.1016/j.jep.2012.03.023 22465145

[18] Ochiai W, Kobayashi H, Kitaoka S, et al. Effect of the active ingredient of *Kaempferia parviflora*, 5,7-dimethoxyflavone, on the pharmacokinetics of midazolam. J Nat Med. 2018; 72(3): 607–614. 10.1007/s11418-018-1184-z 29550915

[19] Guo LQ, Fukuda K, Ohta T, Yamazoe Y. Role of furanocoumarin derivatives on grapefruit juice-mediated inhibition of human CYP3A activity. Drug Metab Dispos. 2000; 28(7): 766–771. 10859150

[20] Wrighton SA, Ring BJ. Inhibition of human CYP3A catalyzed 1′-hydroxy midazolam formation by ketoconazole, nifedipine, erythromycin, cimetidine, and nizatidine. Pharm Res. 1994; 11(6): 921–924. 10.1023/A:1018906614320 7937537

[21] Nishimura Y, Kurata N, Sakurai E, Yasuhara H. Inhibitory effect of antituberculosis drugs on human cytochrome P450-mediated activities. J Pharmacol Sci. 2004; 96(3): 293–300. 10.1254/jphs.FP0040296 15528841

[22] Mandema JW, Tukker E, Danhof M. Pharmacokinetic-pharmacodynamic modelling of the EEG effects of midazolam in individual rats: influence of rate and route of administration. Br J Pharmacol. 1991; 102(3): 663–668. 10.1111/j.1476-5381.1991.tb12230.x 1364836 PMC1917918

[23] Saito Y, Nishimura Y, Kurata N, Iwase M, Aoki K, Yasuhara H. *In vivo* inhibition of CYP3A-mediated midazolam metabolism by anchusan in rats. J Pharmacol Sci. 2011; 115(3): 399–407. 10.1254/jphs.10277FP 21358120

[24] Toshima H, Sanbe T, Kizaki J, et al. Inhibition of CYP3A4-mediated midazolam metabolism by repeat oral administration of anchusan in humans. J Showa Univ Soc. 2013; 73(2): 120–128.

[25] Tabata K, Yamaoka K, Kaibara A, Suzuki S, Terakawa M, Hata T. Moment analysis program available on Microsoft Excel®. Drug Metabolism and Pharmacokinetics. 1999; 14(4): 286–293. 10.2133/dmpk.14.286

[26] Yamaoka K, Tanigawara Y, Nakagawa T, Uno T. A pharmacokinetic analysis program (multi) for microcomputer. J Pharmacobiodyn. 1981; 4(11): 879–885. 10.1248/bpb1978.4.879 7328489

[27] Takara K, Ohnishi N, Horibe S, Yokoyama T. Expression profiles of drug-metabolizing enzyme CYP3A and drug efflux transporter multidrug resistance 1 subfamily mRNAS in small intestine. Drug Metab Dispos. 2003; 31(10): 1235–1239. 10.1124/dmd.31.10.1235 12975332

[28] Walle UK, Walle T. Bioavailable flavonoids: cytochrome P450-mediated metabolism of methoxyflavones. Drug Metab Dispos. 2007; 35(11): 1985–1989. 10.1124/dmd.107.016782 17709371

[29] Obach RS, Walsky RL, Venkatakrishnan K. Mechanism-based inactivation of human cytochrome p450 enzymes and the prediction of drug-drug interactions. Drug Metab Dispos. 2007; 35(2): 246–255. 10.1124/dmd.106.012633 17093004

[30] Tassaneeyakul W, Guo LQ, Fukuda K, Ohta T, Yamazoe Y. Inhibition selectivity of grapefruit juice components on human cytochromes P450. Arch Biochem Biophys. 2000; 378(2): 356–363. 10.1006/abbi.2000.1835 10860553

[31] Mekjaruskul C, Jay M, Sripanidkulchai B. Pharmacokinetics, bioavailability, tissue distribution, excretion, and metabolite identification of methoxyflavones in Kaempferia parviflora extract in rats. Drug Metab Dispos. 2012; 40(12): 2342–2353. 10.1124/dmd.112.047142 22961680

[32] Matsubara T, Kim HJ, Miyata M, Shimada M, Nagata K, Yamazoe Y. Isolation and characterization of a new major intestinal CYP3A form, CYP3A62, in the rat. J Pharmacol Exp Ther. 2004; 309(3): 1282–1290. 10.1124/jpet.103.061671 15004215

[33] Ghosal A, Satoh H, Thomas PE, Bush E, Moore D. Inhibition and kinetics of cytochrome P4503A activity in microsomes from rat, human, and cdna-expressed human cytochrome P450. Drug Metab Dispos. 1996; 24(9): 940–947. 8886602

[34] Kobayashi K, Urashima K, Shimada N, Chiba K. Substrate specificity for rat cytochrome P450 (CYP) isoforms: screening with cDNA-expressed systems of the rat. Biochem Pharmacol. 2002; 63(5): 889–896. 10.1016/S0006-2952(01)00843-7 11911841

